# Sex differences in the association between Life’s Essential 8 and serum anti-aging Klotho protein levels: a cross-sectional analysis in middle-aged to older adults

**DOI:** 10.3389/fragi.2025.1458571

**Published:** 2025-05-30

**Authors:** Jing Zeng, Tingting Zhang, Yan Yang, Jinjing Wang, Xiaojing Fan, Qiaomin Wang, Xuan Wang, Haoxian Tang, Yi Fang

**Affiliations:** ^1^ Department of Endocrinology, Fifth Medical Center of Chinese PLA General Hospital, Beijing, China; ^2^ Shantou University Medical College, Shantou, China; ^3^ Department of Cardiology, The First Affiliated Hospital of Shantou University Medical College, Shantou, China

**Keywords:** sex differences, Life's Essential 8, cardiovascular health, Klotho, aging, NHANES

## Abstract

**Background:**

This study explores the association between cardiovascular health metrics (Life’s Essential 8, LE8) and serum anti-aging Klotho protein levels among American adults aged 40–79, with a focus on sex-specific differences.

**Methods:**

Utilizing data from the 2007–2016 National Health and Nutrition Examination Survey (NHANES), we applied weighted multivariable regression analyses, restricted cubic spline (RCS) modeling, and subgroup analyses to investigate the association between Life’s Essential 8 (LE8) scores—including Health Behaviors and Health Factors scores—and serum Klotho concentrations, with a focus on gender differences.

**Results:**

Our study encompassed 9,534 participants, including 4,946 females and 4,588 males. Weighted multivariable regression analyses revealed that only females exhibited a positive association between Life’s Essential 8 (LE8) scores and serum Klotho protein levels. Specifically, a 10-point increase in LE8 scores resulted in an elevation of Klotho levels by 17.61 pg/mL (95% CI: 9.53–25.69). Similarly, a 10-point increase in Health Behaviors scores increased Klotho levels by 5.7 pg/mL (95% CI: 0.14–11.26), and a 10-point increase in Health Factors scores was associated with a rise in Klotho levels by 14.6 pg/mL (95% CI: 7.70–21.51). Sensitivity analyses showed a significant positive trend in Klotho levels correlating with improvements in cardiovascular health (CVH) levels among females (β = 72.9, 95% CI: 35.91–109.88; p for trend <0.001), consistent across both Health Behaviors and Health Factors scores. No similar trends were noted in males. In females, the dose-response relationship between LE8 scores and Health Factors scores displayed nonlinear patterns, whereas Health Behaviors scores showed linear patterns. Optimal threshold was identified at 65 for LE8 score. These results were consistent across various demographic and health statuses, emphasizing the sex-specific influence of LE8 scores on serum Klotho levels. This study underscores the importance of considering gender differences in cardiovascular health and its impact on anti-aging protein levels.

**Conclusion:**

In the present study, LE8 scores and its sub-scales were positively related to serum Klotho level in middle-aged and elderly women. This study provides new evidence of sex differences in the association between LE8 scores and serum anti-aging Klotho protein levels.

## Introduction

Aging is marked by the decline of essential physiological functions necessary for survival and fertility (Wang and Sun, 2009). There is significant diversity in the aging processes between genders, with women generally exhibiting longer lifespans and lower biological ages as indicated by molecular biomarkers (Hagg and Jylhava, 2021).

In 1997, Kuro-o et al. identified the Klotho gene, named after the Greek goddess who spins the thread of life, and highlighted its significant anti-aging effects (Kuro-o et al., 1997). Studies in both animal models and humans have demonstrated that the Klotho gene possesses anti-aging properties. Mice with reduced expression of this gene show a shortened lifespan and early aging signs (Kuro-o et al., 1997), whereas its overexpression has been found to extend lifespan (Masuda et al., 2005). In humans, the Klotho gene has been linked to increased longevity and potentially lowers the risk of several aging-related conditions, such as cardiovascular diseases (CVD) (Kim et al., 2006), disorders of glucose metabolism (Shimoyama et al., 2009), and cognitive impairment (Wolf et al., 2010; Hao et al., 2016; Cararo-Lopes et al., 2017), among others. Despite these findings, the understanding of gender-specific differences in the expression and function of the Klotho gene remains limited.

Cardiovascular health (CVH) is known to manifest differently between men and women, yet the underlying causes of these sex-specific differences during aging are not well understood (Ji et al., 2022). In 2022, the American Heart Association (AHA) revised the Life’s Simple 7 (LS7) into the enhanced Life’s Essential 8 (LE8) to better quantify CVH levels and improve overall population health (Lloyd-Jones et al., 2022). The LE8 scoring system considers differences between individuals as well as variations within the same individual over time. Extensive research has shown a significant, progressive negative correlation between LE8 scores and CVD, all-cause mortality, and various non-CVD outcomes (Wang et al., 2023; Sun J. et al., 2023; Sun Y. et al., 2023). Additionally, recent studies have shown that higher LE8 scores and its subscale scores are strongly inversely related to markers of biological aging in American adults aged 20 and older (Zhang et al., 2023).

Although the relationship between the LE8 score and aging is documented, the impact of sex differences on this association, particularly with serum anti-aging Klotho protein levels, remains poorly understood. This study aims to explore these sex differences in the relationship between LE8 score and serum Klotho levels among US adults aged 40 to 79.

## Methods

### Study design and participants

This study sourced its data from the National Health and Nutrition Examination Survey (NHANES), a nationally representative cross-sectional survey conducted by the National Center for Health Statistics (NCHS). NHANES employs a stratified, multistage probability sampling method to collect health and nutritional data from the non-institutionalized civilian population of the United States. This survey has been approved by the National Center for Health Statistics Research Ethics Review Board. Informed consent was obtained from all participants. Further details on the survey methodology are available on the NCHS website at https://www.cdc.gov/nchs/nhanes/.

**FIGURE 1:**
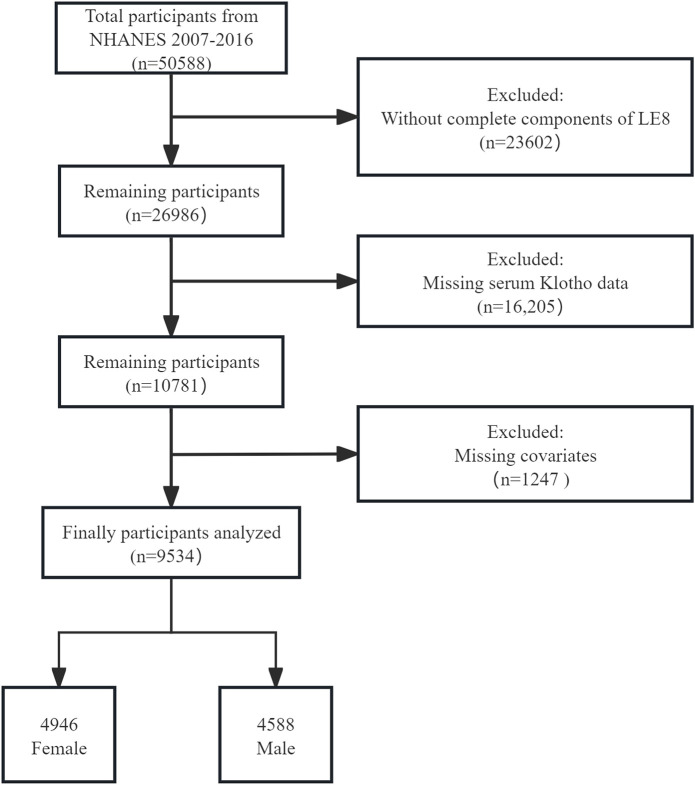
Flowchart of participant selection. NHANES, National Health and Nutrition Examination Survey.

This study utilized standardized in-home interviews, physical examinations, and laboratory tests conducted at mobile examination centers to assess the nutritional and physical health of participants. Data were collected from 50,588 participants across the 2007–2016 NHANES cycles. Exclusions were based on incomplete LE8 data for 23,602 individuals, missing serum Klotho data for 16,205, and missing covariate data for 1,247. Following these criteria, 9,534 participants were eligible for analysis, as depicted in Figure 1. The study adhered to the Strengthening the Reporting of Observational Studies in Epidemiology (STROBE) guidelines to ensure rigorous observational research standards (von Elm et al., 2007).

### Definitions and metrics for LE8 and CVH levels

In 2022, the AHA updated the LS7 framework to LE8 to better quantify CVH (Lloyd-Jones et al., 2022). The LE8 framework includes four health behaviors—diet, physical activity (PA), nicotine exposure, and sleep health—and four health factors—body mass index (BMI), blood lipids, blood glucose, and blood pressure. The scoring criteria for each LE8 metric are derived from methodologies outlined in prior research (Cai et al., 2023; Chen et al., 2023; Wang et al., 2022). Diet scores were assessed during in-person 24-h dietary recall interviews using the Dietary Approaches to Stop Hypertension and the US Department of Agriculture’s Automated Multiple-Pass Method to quantify food and beverage intake. PA was measured by the weekly duration participants engaged in activities like walking, biking, household chores, yard work, and recreational activities. Nicotine exposure was evaluated through self-reports of cigarette smoking or use of inhaled nicotine systems, as well as exposure to indoor smokers. Sleep health was gauged by average nightly sleep duration. BMI was calculated as weight in kilograms divided by the square of height in meters. Blood lipids were determined by calculating non-high-density lipoprotein cholesterol from total and high-density lipoprotein levels. Average blood pressure (BP) was calculated by excluding the first measurement, unless it was the only measurement available. Blood glucose levels were assessed using fasting plasma glucose or hemoglobin A1c levels. Additionally, the use of medications for managing hypercholesterolemia, hypertension, and diabetes was documented based on self-reports.

In the LE8 framework, each metric has a scoring algorithm that ranges from 0 to 100 points, used to create a composite CVH score. This composite score, also ranging from 0 to 100, is the unweighted average of all component scores. According to the AHA, LE8 scores of 80 or above are classified as high CVH, scores between 50 and 79 as moderate CVH, and scores below 50 signify low CVH (Lloyd-Jones et al., 2022). In our study, we used these scoring thresholds to categorize participants’ health behaviors and factors, specifically to examine their association with serum Klotho levels.

### Serum Klotho levels

Serum samples were preserved at −80°C at the Centers for Disease Control and Prevention in Atlanta, GA. Between 2019 and 2020, these samples were transported on dry ice to the Northwest Lipid Metabolism and Diabetes Research Laboratories at the University of Washington in Seattle, WA, where Klotho quantification was performed. Klotho analysis was conducted using an enzyme-linked immunosorbent assay (ELISA) kit provided by IBL International, Japan (Yamazaki et al., 2010). The results were transmitted automatically from the assay instrument to the laboratory’s Oracle management system and reviewed by the regional supervisor. Each serum sample was analyzed in duplicate, and the average of the two readings was calculated as per the manufacturer’s instructions. Duplicate tests that varied by more than 10% were flagged for reanalysis. The detection limit for Klotho was established at 6 pg/mL, and as all measured values were within this threshold, there was no need for data imputation for any of the samples (Wu et al., 2023).

### Covariates

Building upon prior research, our study included several covariates: sex, age, race/ethnicity, marital status, educational level, poverty income ratio (PIR), drinking status, and history of CVD, chronic kidney disease (CKD), and cancer (Chen et al., 2023; Kadier et al., 2024). Age was categorized into two groups: 40–59 years and 60–79 years, based on baseline assessments. Race/ethnicity was classified into five categories: Mexican American, non-Hispanic black, non-Hispanic white, other Hispanic, and other. Marital status was defined as either married/living with a partner or living alone. Educational levels were segmented into three groups: less than high school, high school or equivalent, and above than high school. The PIR was divided into three ranges: low (≤1.30), middle (1.31–3.50), and high (>3.50). Drinking status, as reported by participants, was categorized as never (<12 lifetime drinks), former (≥12 drinks in 1 year but not the last year, or stopped after a lifetime consumption of ≥12 drinks), mild (up to 1 drink per day for females and 2 for males), moderate (up to 2 drinks per day for females and 3 for males), or heavy (≥3 drinks per day for females and ≥4 for males). The history of CVD included any self-reported diagnosis of heart failure, coronary heart disease, angina, heart attack, or stroke. CKD was identified by an eGFR ≤60 mL/min/1.73 m^2^ or a urinary albumin/creatinine ratio ≥30 mg/g (Cui et al., 2023). Cancer status was determined by self-reported physician diagnoses.

### Statistical analysis

Following the NHANES analytical guidelines, we incorporated the complex sampling design and applied mobile examination center sample weights (WTMEC2YR×1/5) in our analysis. For further details on the guidelines, see the NHANES tutorials (https://wwwn.cdc.gov/nchs/nhanes/tutorials/default.aspx). Baseline characteristics stratified by sex were described using means ± standard error (SE) for continuous variables and percentage frequencies (%) for categorical variables. We examined sex differences using chi-squared tests with Rao & Scott’s second-order correction and Wilcoxon rank-sum tests, both adjusted appropriately for the survey’s complex design.

Associations between LE8 score, CVH levels, and serum Klotho levels were analyzed separately for males and females using multivariable weighted linear regression models, which provided regression coefficients (β) and 95% confidence intervals (CI) for every 10-point increase in LE8 score. The models evaluated CVH levels, categorized as low, moderate, and high, with low CVH serving as the reference category. Additionally, the relationships between the health behaviors score, the health factors score, and serum Klotho levels were assessed using weighted linear regression, estimating β and 95% CI for each 10-point score increment across CVH levels, with low CVH as the reference. The analysis employed three models: Model 1 was unadjusted; Model 2 was adjusted for demographic and lifestyle factors including age, race/ethnicity, marital status, educational level, poverty income ratio (PIR), and drinking status; Model 3 additionally included adjustments for a history of CVD, CKD, and cancer.

In the sex groups demonstrating positive associations, we conducted further analyses to explore non-linear relationships between LE8 score, health behaviors score, health factors score, and serum Klotho levels. These analyses utilized restricted cubic splines (RCS) within Model 3, which was comprehensively adjusted for demographic and health-related variables.

Within the sex groups exhibiting positive associations, we employed stratified multivariate linear regression to explore the nuanced relationships between LE8 score, health behaviors score, health factors score, and serum Klotho levels. Subgroup analyses were performed, stratifying by various demographic and health status variables, including age (40–59 years and 60–79 years), race (Mexican American, non-Hispanic black, non-Hispanic white, other Hispanic, and other), marital status (married or living with a partner, and living alone), educational level (less than high school, high school or equivalent, and above high school), PIR (low, middle, and high), drinking status (never, former, mild, moderate, and heavy), history of CVD (no and yes), CKD (no and yes), and cancer history (no and yes).

All statistical analyses were conducted using R software (version 4.3.2) and its associated packages, available from The R Foundation at www.R-project.org. Additionally, Free Statistics software (version 1.9.2; Beijing Free Clinical Medical Technology Co., Ltd.) was extensively utilized in our analysis. Statistical significance was defined as a two-sided p-value of less than 0.05. Data analysis was conducted from February to June 2024.

## Results

### Baseline characteristics of study participants

Table 1 presented the baseline characteristics of 9,534 participants, including 4,946 females and 4,588 males. These participants represent an extrapolated demographic of 83,613,883 U.S. adults aged 40–79. The weighted average age of the participants was 56.28 ± 0.18 years. The mean score for LE8 was 66.44 ± 0.34. The distribution of CVH levels was as follows: 1,592 participants had low CVH, 6,552 had moderate CVH, and 1,390 had high CVH. The average serum Klotho protein concentration was 842.39 ± 5.48 pg/mL. Female participants exhibited significantly higher levels (860.79 ± 6.94 pg/mL) compared to males (821.83 ± 6.65 pg/mL, *p* < 0.001). Further baseline characteristics according to CVH levels were detailed in Supplementary Table S1. To further validate the results, the baseline characteristics stratified by sex, utilizing weighted, imputed data through multiple imputation were illustrated in Supplementary Table S2.

**TABLE 1:** Baseline characteristics stratified by sex in weighted^a^.

Characteristic	Overall N = 9534	Female N = 4946	Male N = 4588	*p*-value
Age, Mean (SE)	56.28 (0.18)	56.39 (0.20)	56.16 (0.21)	0.25
Age, n (%)				0.54
40–59	5,143 (62.45%)	2,727 (62.13%)	2,416 (62.81%)	
60–79	4,391 (37.55%)	2,219 (37.87%)	2,172 (37.19%)	
Race/ethnicity, n (%)^b^				0.020
Mexican American	1,356 (5.50%)	700 (5.27%)	656 (5.76%)	
Non-Hispanic Black	995 (3.93%)	549 (3.93%)	446 (3.93%)	
Non-Hispanic White	4,599 (77.14%)	2,365 (76.78%)	2,234 (77.53%)	
Other Hispanic	1,846 (8.34%)	966 (9.02%)	880 (7.58%)	
Other Race^c^	738 (5.10%)	366 (5.00%)	372 (5.20%)	
Marital status, n (%)				<0.001
Married or living with partner	6,237 (71.41%)	2,867 (65.61%)	3,370 (77.90%)	
Living alone	3,297 (28.59%)	2,079 (34.39%)	1,218 (22.10%)	
Education level, n (%)				<0.001
Less than high school	4,458 (36.49%)	2,261 (35.84%)	2,197 (37.21%)	
High school or equivalent	2,716 (30.75%)	1,521 (32.81%)	1,195 (28.44%)	
Above high school	2,360 (32.77%)	1,164 (31.35%)	1,196 (34.35%)	
PIR, n (%)				<0.001
Low	2,668 (15.68%)	1,453 (16.72%)	1,215 (14.52%)	
Middle	3,461 (32.85%)	1,831 (34.26%)	1,630 (31.27%)	
High	3,405 (51.47%)	1,662 (49.01%)	1,743 (54.21%)	
Drinking status, n (%)				<0.001
Never	1,249 (9.61%)	947 (13.12%)	302 (5.69%)	
Former	2,071 (17.88%)	1,016 (17.10%)	1,055 (18.76%)	
Mild	3,426 (40.70%)	1,550 (36.47%)	1,876 (45.42%)	
Moderate	1,370 (16.81%)	876 (21.30%)	494 (11.78%)	
Heavy	1,418 (15.00%)	557 (12.01%)	861 (18.34%)	
CVD history, n (%)^d^				<0.001
No	8,291 (89.59%)	4,427 (91.75%)	3,864 (87.19%)	
Yes	1,243 (10.41%)	519 (8.25%)	724 (12.81%)	
CKD, n (%)				0.84
No	8,030 (87.53%)	4,214 (87.61%)	3,816 (87.44%)	
Yes	1,504 (12.47%)	732 (12.39%)	772 (12.56%)	
Cancer history, n (%)				0.23
No	8,333 (85.79%)	4,318 (85.26%)	4,015 (86.38%)	
Yes	1,201 (14.21%)	628 (14.74%)	573 (13.62%)	
LE8, Mean (SE)	66.44 (0.34)	67.02 (0.42)	65.79 (0.35)	<0.001
CVH3, n (unweighted) (%)				<0.001
Low	1,592 (12.97%)	860 (13.76%)	732 (12.09%)	
Moderate	6,552 (67.77%)	3,240 (64.15%)	3,312 (71.82%)	
High	1,390 (19.26%)	846 (22.10%)	544 (16.09%)	
Health behaviors score, Mean (SE)	67.49 (0.45)	67.53 (0.56)	67.46 (0.47)	0.62
Diet score, Mean (SE)	42.75 (0.64)	44.73 (0.79)	40.54 (0.71)	<0.001
PA score, Mean (SE)	70.85 (0.78)	66.48 (1.01)	75.73 (0.91)	<0.001
Nicotine exposure score, Mean (SE)	72.24 (0.68)	74.45 (0.76)	69.78 (0.88)	<0.001
Sleep health score, Mean (SE)	84.13 (0.37)	84.45 (0.48)	83.78 (0.45)	0.051
Health factors score, Mean (SE)	65.39 (0.34)	66.52 (0.46)	64.13 (0.36)	<0.001
BMI score, Mean (SE)	57.92 (0.55)	58.42 (0.79)	57.36 (0.63)	0.065
Blood lipids score, Mean (SE)	58.39 (0.44)	59.50 (0.56)	57.15 (0.62)	0.004
Blood glucose score, Mean (SE)	81.51 (0.45)	82.70 (0.58)	80.18 (0.54)	<0.001
BP score, Mean (SE)	63.74 (0.49)	65.44 (0.56)	61.83 (0.71)	<0.001
Klotho, Mean (SE), (pg/mL)	842.39 (5.48)	860.79 (6.94)	821.83 (6.65)	<0.001

^a^ All means and SEs for continuous variables and percentages for categorical variables were weighted.

^b^ Race and ethnicity were self-reported.

^c^ Includes multiracial participants. SE, standard error; PIR, poverty income ratio; CVD, cardiovascular disease.

^d^ Includes congestive heart failure, coronary heart disease, angina, heart attack, and stroke.

CKD, chronic kidney disease; LE8, life’s essential 8; CVH, cardiovascular health score; PA, physical activity; BMI, body mass index; BP, blood pressure.

### Multivariate logistic regression analysis of the LE8 score and serum Klotho levels

As detailed in Table 2, there was a positive association between LE8 score and serum Klotho levels in females. Specifically, every 10-point increment in LE8 score was associated with a significant increase in serum Klotho levels (β: 20.6, 95% CI: 13.79–27.42, *p* < 0.001) in Model 1. When stratified by CVH levels—low, moderate, and high—participants in the high CVH level showed the highest increase in serum Klotho levels (β: 90.83, 95% CI: 57.21–124.44, *p* < 0.001) compared to those at the low CVH level. Although no significant difference was observed for the moderate CVH level compared to the low level, the trend in the relationship between LE8 score/CVH level and serum Klotho levels remained statistically significant (p for trend <0.001). This trend persisted across Model 2 and Model 3. Additionally, the health behaviors and health factors scores were positively correlated with serum Klotho levels. Each 10-point increase in health behaviors and health factors scores was associated with rises in serum Klotho levels (β: 8.71, 95% CI: 3.87–13.56, *p* < 0.001 and β: 16.64, 95% CI: 10.43–22.85, *p* < 0.001, respectively) in Model 1. When analyzed categorically, serum Klotho levels increased significantly at the high level of both scores compared to the low level in Model 1 (health behaviors score: β: 55.93, 95% CI: 26.66–85.20, *p* < 0.001; health factors score: β: 79.24, 95% CI: 47.87–110.61, *p* < 0.001). The moderate levels did not exhibit significant differences from the low level, but the overall trend was significant (*p* for trend <0.001). This trend was consistent across Model 2 and Model 3. In Supplementary Table S3, 4, the fully adjusted weighted linear regression models revealed significant positive associations between BMI score, blood lipids score, and BP score of LE8 components with serum Klotho levels in females.

**TABLE 2:** Associations of LE8, health behaviors, and health factors scores with serum Klotho in females.

Variables	Model 1^a^		Model 2^b^		Model 3^c^	
	β (95% CI)	*p* Value	β (95% CI)	*p* Value	β (95% CI)	*p* Value
LE8 score						
Per 10 points increase	20.6 (13.79–27.42)	<0.001	19.33 (11.44–27.22)	<0.001	17.61 (9.53–25.69)	<0.001
Low CVH	1(Ref)		1(Ref)		1(Ref)	
Moderate CVH	21.17 (−9.72–52.06)	0.176	22.71 (−8.21–53.63)	0.147	15.81 (−15.16–46.78)	0.311
High CVH	90.83 (57.21–124.44)	<0.001	81.53 (45.35–117.72)	<0.001	72.9 (35.91–109.88)	<0.001
Trend.test		<0.001		<0.001		0.001
Health behaviors score						
Per 10 points increase	8.71 (3.87–13.56)	<0.001	6.67 (1.08–12.25)	0.02	5.7 (0.14–11.26)	0.045
Low (0–49)	1(Ref)		1(Ref)		1(Ref)	
Moderate (50–79)	14.93 (−8.24–38.10)	0.203	8.62 (−16.74–33.99)	0.499	5.13 (−20.34–30.60)	0.689
High (80–100)	55.93 (26.66–85.20)	<0.001	46.31 (12.97–79.64)	0.007	40.46 (7.06–73.85)	0.018
Trend.test		<0.001		0.004		0.011
Health factors score						
Per 10 points increase	16.64 (10.43–22.85)	<0.001	15.85 (9.09–22.61)	<0.001	14.6 (7.70–21.51)	<0.001
Low (0–49)	1(Ref)		1(Ref)		1(Ref)	
Moderate (50–79)	2.13 (−17.85–22.12)	0.832	4.81 (−15.39–25.00)	0.636	−2.34 (−23.46–18.78)	0.825
High (80–100)	79.24 (47.87–110.61)	<0.001	73.84 (40.33–107.35)	0.002	66.86 (32.98–100.74)	<0.001
Trend.test		<0.001		0.001		<0.001

^a^ Crude model.

^b^ Adjusted for age, race/ethnicity, marital status, educational level, PIR, and drinking status.

^c^ Adjusted for age, race/ethnicity, marital status, educational level, PIR, drinking status; CVD, history; CKD, and cancer history.

As shown in Table 3, the associations between LE8 score, health behaviors score, health factors score, and serum Klotho levels were not statistically significant in males across all models, whether considered as continuous or categorical variables.

**TABLE 3:** Associations of LE8, health behaviors, and health factors scores with serum Klotho in males.

Variables	Model 1^a^		Model 2^b^		Model 3^c^	
	β (95% CI)	*p* Value	β (95% CI)	*p* Value	β (95% CI)	*p* Value
LE8 score						
Per 10 points increase	3.79 (−3.27–10.85)	0.288	0.18 (−8.23–8.59)	0.966	−0.98 (−9.49–7.53)	0.819
Low CVH	1(Ref)		1(Ref)		1(Ref)	
Moderate CVH	4.97 (−26.41–36.35)	0.753	2.85 (−29.72–35.42)	0.862	0.54 (−31.44–32.52)	0.973
High CVH	23.88 (−9.55–57.30)	0.159	10.51 (−28.01–49.02)	0.588	6.4 (−31.95–44.74)	0.74
Trend.test		0.147		0.58		0.726
Health behaviors score						
Per 10 points increase	1.67 (−3.43–6.78)	0.516	−0.04 (−5.82–5.73)	0.988	−0.24 (−6.04–5.56)	0.934
Low (0–49)	1(Ref)		1(Ref)		1(Ref)	
Moderate (50–79)	−8.93 (−40.03–22.17)	0.569	−12.51 (−44.26–19.25)	0.434	−12.68 (−44.24–18.87)	0.425
High (80–100)	9.19 (−19.51–37.88)	0.526	0.5 (−31.15–32.16)	0.975	−0.68 (−32.60–31.23)	0.966
Trend.test		0.345		0.783		0.849
Health factors score						
Per 10 points increase	2.78 (−4.35–9.91)	0.44	0.26 (−7.13–7.65)	0.944	−0.86 (−8.36–6.64)	0.819
Low (0–49)	1(Ref)		1(Ref)		1(Ref)	
Moderate (50–79)	−0.8 (−31.85–30.25)	0.832	−4.96 (−35.79–25.87)	0.749	−9.64 (−41.07–21.79)	0.542
High (80–100)	8.07 (−33.64–49.78)	0.701	−4.38 (−46.80–38.04)	0.837	−10.08 (−53.62–33.46)	0.645
Trend.test		0.699		0.837		0.648

^a^ Crude model.

^b^ Adjusted for age, race/ethnicity, marital status, educational level, PIR, and drinking status.

^c^ Adjusted for age, race/ethnicity, marital status, educational level, PIR, drinking status; CVD, history; CKD, and cancer history.

The associations of LE8 score, health behaviors score, and health factors score with serum Klotho levels in females and males, analyzed using multiple imputation for missing data, are displayed in Supplementary Table S5, 6, respectively. Sensitivity analyses further corroborated these findings. The associations of LE8 score, health behaviors score, and health factors score with serum Klotho concentrations for all participants are presented in Supplementary Table S7.

### Analysis of dose-response relationships between LE8 score, health behaviors score, health factors score, and serum Klotho levels in females

**FIGURE 2:**
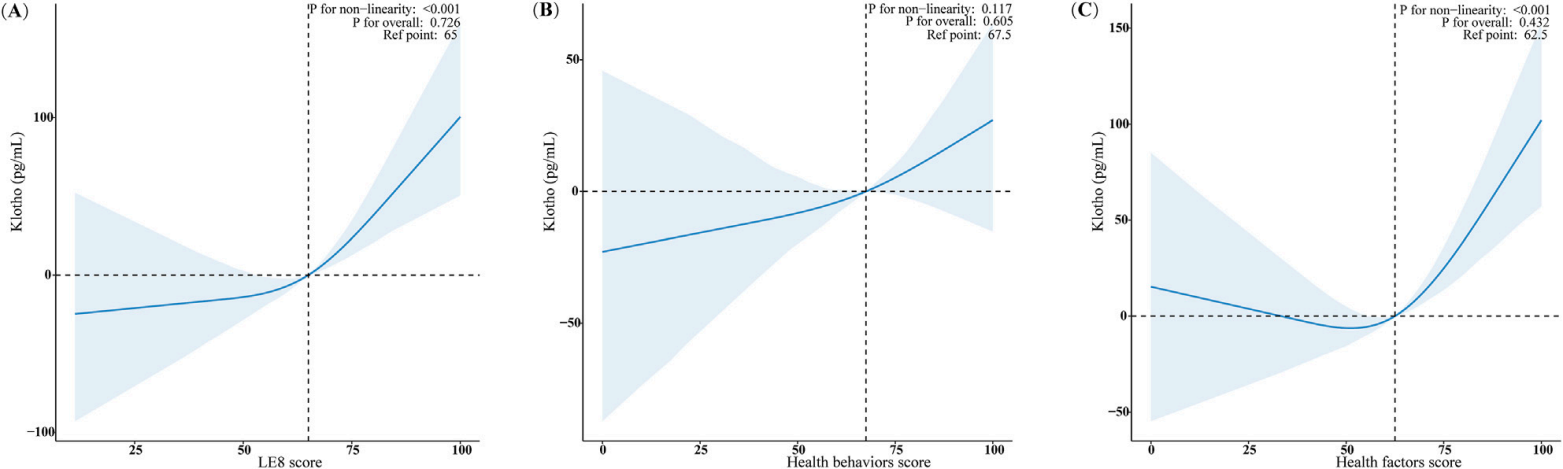
Analysis of dose-response relationships between LE8 score, Health behaviors and Health factors score, and serum Klotho level in females. **(A)** LE8 score; **(B)** Health behaviors score; **(C)** Health factors score. The RCS model adjusted for age, race/ethnicity, marital status, educational level, PIR, drinking status, CVD, CKD, and cancer history. Only 99% of the data is shown.

Using restricted cubic spline (RCS) regression and adjusting for all covariates, we observed significant positive non-linear relationships between the LE8 score, the health factors score, and serum Klotho levels in females, with *p*-values for non-linearity below 0.001 (Figure 2A,C). The relationship between the LE8 score and serum Klotho levels exhibited a “J”-shaped curve, while the association between the health factors score and serum Klotho levels displayed a “U”-shaped curve. The beneficial threshold for the LE8 score was identified at a score of 65, where the estimated regression coefficient (β) reaches zero. Conversely, the analysis demonstrated a linear relationship between the health behaviors score and serum Klotho levels in females, as evidenced by non-significant non-linearity (*p* = 0.117; Figure 2B).

### Subgroup analyses of LE8 scores, health behaviors scores, health factors scores, and serum Klotho levels in females

**FIGURE 3:**
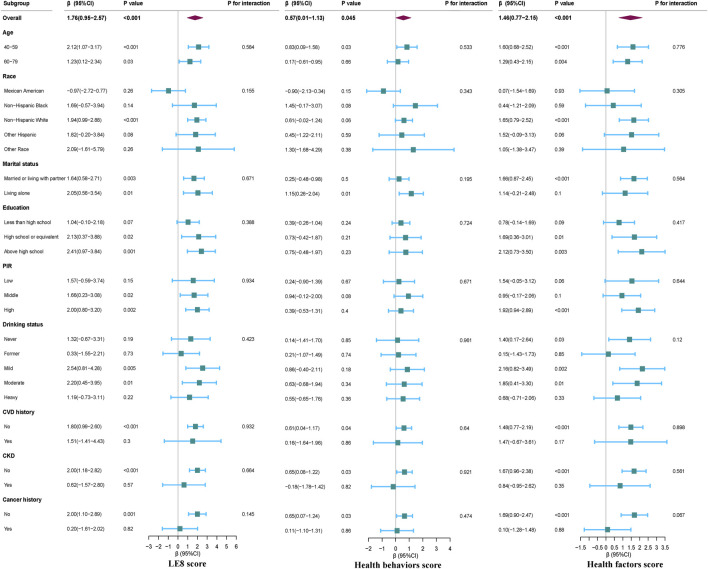
Subgroup analyses for the association between LE8 Score, Health behaviors and Health factors score, and serum Klotho level in females. Except for the stratification component itself, each stratification factor was adjusted for all other variables (age, race, education level, family income, smoking status, drinking status, physical activity, UA, TC, LDL-C, diabetes and CVD).

Our subgroup analyses, as illustrated in Figure 3, demonstrate positive associations between LE8 score, health behaviors scores, and health factors scores with serum Klotho levels in females, after adjusting for multiple latent variables (*p* < 0.05). No significant interaction effects were detected within these subgroups (all *p*-values for interaction >0.05).

Subgroup analyses on the association between LE8 score and its subscales with serum Klotho levels for all study participants are presented in Supplementary Figure S1. These additional subgroup analyses reveal that sex modifies the relationship between LE8 score, health behaviors score, health factors scores, and serum Klotho levels (all *p*-values for interaction <0.05), emphasizing the variable impact of these associations across different demographic groups.

## Discussion

In this population-based study, we observed that the LE8 score and health behaviors/health factors scores were significantly associated with increased serum Klotho levels in females, with no corresponding associations in males. RCS regression analyses showed a nonlinear relationship for the LE8 score, with beneficial thresholds established at 65. There were no significant interactions between serum Klotho concentrations and these scores across various subgroups of females. These findings highlight the importance of optimal cardiovascular health in females for maintaining higher levels of the anti-aging Klotho protein. This study is the first to investigate sex differences in the relationship between LE8 score and serum Klotho levels in a substantial population.

Recent studies have demonstrated that cardiovascular health (CVH), together with health behaviors and factors, plays a critical role in aging and modulating Klotho levels. Research by Zhang et al. identified negative correlations between the Life’s Essential 8 (LE8) score and its subscales (health behaviors and health factors) with biological aging, with health factors, especially blood glucose and blood pressure, showing the strongest negative correlations (Zhang et al., 2023). Conversely, Wu *et al.* found a positive link between adherence to the Mediterranean Diet and higher Klotho levels, unlike other diets such as low-carbohydrate and low-fat, which did not show similar associations (Wu et al., 2022). Further exploring the impact of lifestyle on Klotho, F.J. Amaro-Gahete *et al.* suggested that exercise-induced body composition changes—specifically reductions in fat mass and increases in lean mass—might enhance plasma Klotho levels, illustrating a possible mechanistic link (Amaro-Gahete et al., 2019). Additionally, cessation of smoking was significantly correlated with decreased serum Klotho levels, as noted by Yoko Kamizono et al., proposing that Klotho might respond compensatorily to the oxidative stress induced by smoking (Kamizono et al., 2018). Sol Mochón-Benguigui *et al.* reported a direct correlation between good subjective sleep quality and higher S-Klotho levels in sedentary middle-aged adults (Mochon-Benguigui et al., 2020), while Cui *et al.* described a nonlinear relationship between visceral adiposity and serum Klotho, with levels peaking when the visceral adiposity index was below 3.21 (Cui et al., 2023). Cardioprotective interventions also appear to influence Klotho expression. For instance, statin therapy in a White New Zealand rabbit model of atherogenesis markedly reduced inflammation and increased Klotho expression in myocardial tissues (Mylonas et al., 2024). Similarly, the SGLT2 inhibitors, a class of antidiabetic medications, are believed to enhance longevity through mechanisms involving soluble Klotho (Maltese et al., 2023). Furthermore, an extensive cohort study revealed that higher serum Klotho levels were inversely related to pulse pressure, indicating a link with reduced arterial stiffness (Alkalbani et al., 2022). A recent study by Kadier *et al.* examined the association between ideal CVH and serum Klotho protein levels in middle-aged and older populations, emphasizing the importance of maintaining CVH for higher Klotho levels (Kadier et al., 2024). While their findings provide valuable insights into the overall relationship between CVH and Klotho, our study builds on this understanding by specifically focusing on sex differences. We found that the association between LE8 scores and Klotho levels was significant only in women, suggesting a potential sex-specific mechanism underlying this relationship. Our supplementary analyses of the overall population are consistent with Kadier *et al.*'s findings, showing a positive association between CVH metrics and Klotho levels (Supplementary Tables S1, S7 and Supplementary Figure 1). However, our study uniquely identified sex as a critical factor, with women exhibiting a stronger and more consistent association between LE8 scores and Klotho levels. This sex-specific effect may be attributed to hormonal differences, genetic factors, or variations in cardiovascular risk profiles between men and women. This distinct pattern underscores the need to consider sex-specific effects in longevity and anti-aging research. These findings advocate for a nuanced approach in future studies to better understand how gender differences influence CVH and Klotho levels, emphasizing the importance of personalized interventions in aging and disease prevention strategies.

Improved CVH promotes an environment that reduces inflammation and oxidative stress, conditions that typically suppress Klotho expression (Gebreab et al., 2017; Kim et al., 2017). Healthy cardiovascular conditions enable better endothelial function and reduce arterial stiffness, both of which are crucial in preserving heart health and enhancing Klotho production (Epstein and Freundlich, 2022). The protein itself contributes to CVH by increasing nitric oxide availability, which improves vascular tone and flexibility, and by preventing pathological changes such as fibrosis and vascular calcification (Epstein and Freundlich, 2022). Gender differences further modulate these mechanisms. Studies have shown that females typically have higher baseline Klotho levels than males, a disparity often attributed to estrogen’s protective effects on CVH and its potential to upregulate Klotho expression (Chen and Sun, 2021). Estrogen is known to enhance endothelial health and might interact synergistically with Klotho to provide heightened cardiovascular protection in females (Epstein and Freundlich, 2022). Consequently, maintaining cardiovascular health could be particularly beneficial for increasing Klotho levels in women, potentially offering them a greater degree of protection against the cardiovascular declines associated with aging.

Our study offers several advantages, notably utilizing a representative sample of the US population collected through the NHANES between 2007 and 2016. This dataset benefits from a well-designed study protocol that includes extensive quality assurance and quality control measures. As a secondary step, we controlled for confounding covariates to ensure the reliability and broad applicability of our results. Despite these strengths, our study also has limitations. The cross-sectional nature of the study precludes determining the temporal associations between LE8 scores and serum anti-aging Klotho protein levels. Acknowledging this, we recommend that future research exploring sex differences in the association between LE8 and Klotho levels should adopt longitudinal designs. Such studies would allow for tracking changes over time, providing clearer insights into whether elevated LE8 scores precede increases in Klotho protein levels and potentially establishing a causal relationship. Furthermore, the exclusion of a significant number of participants due to missing covariate data might introduce bias. To address this, we employed multiple imputation (MI) techniques to fill gaps in our data and conducted a thorough re-analysis of the imputed dataset. Our sensitivity analysis confirms the consistency of our primary conclusions, enhancing our confidence in the robustness of our findings. Lastly, the study could not account for additional potential confounders such as environmental exposures, genetic factors, and immune factors. This limitation is inherent to cross-sectional studies that rely on previously collected data, potentially lacking the specificity and accuracy of clinical diagnoses. These limitations underscore the need for future longitudinal studies to further investigate these aspects.

## Conclusion

This study establishes a correlation between CVH and the levels of the anti-aging Klotho protein, particularly in females. It identifies an association between the LE8 score and serum Klotho levels, suggesting that improved CVH may be linked to increased Klotho levels, which could potentially mitigate aging and extend longevity. These findings, derived from a substantial and diverse population, underscore the importance of targeted lifestyle interventions and pave the way for personalized medical strategies to combat age-related diseases and enhance quality of life in aging populations.

## Data Availability

The datasets presented in this study can be found in online repositories. The names of the repository/repositories and accession number(s) can be found below: https://www.cdc.gov/nchs/nhanes/.
