# Supplementary figures and images for: Sex differences in the association between Life’s Essential 8 and serum anti-aging Klotho protein levels: a cross-sectional analysis in middle-aged to older adults
Source: Front Aging. 2025 May 30;6:1458571. doi: 10.3389/fragi.2025.1458571 (PMC12162483; doi:10.3389/fragi.2025.1458571)

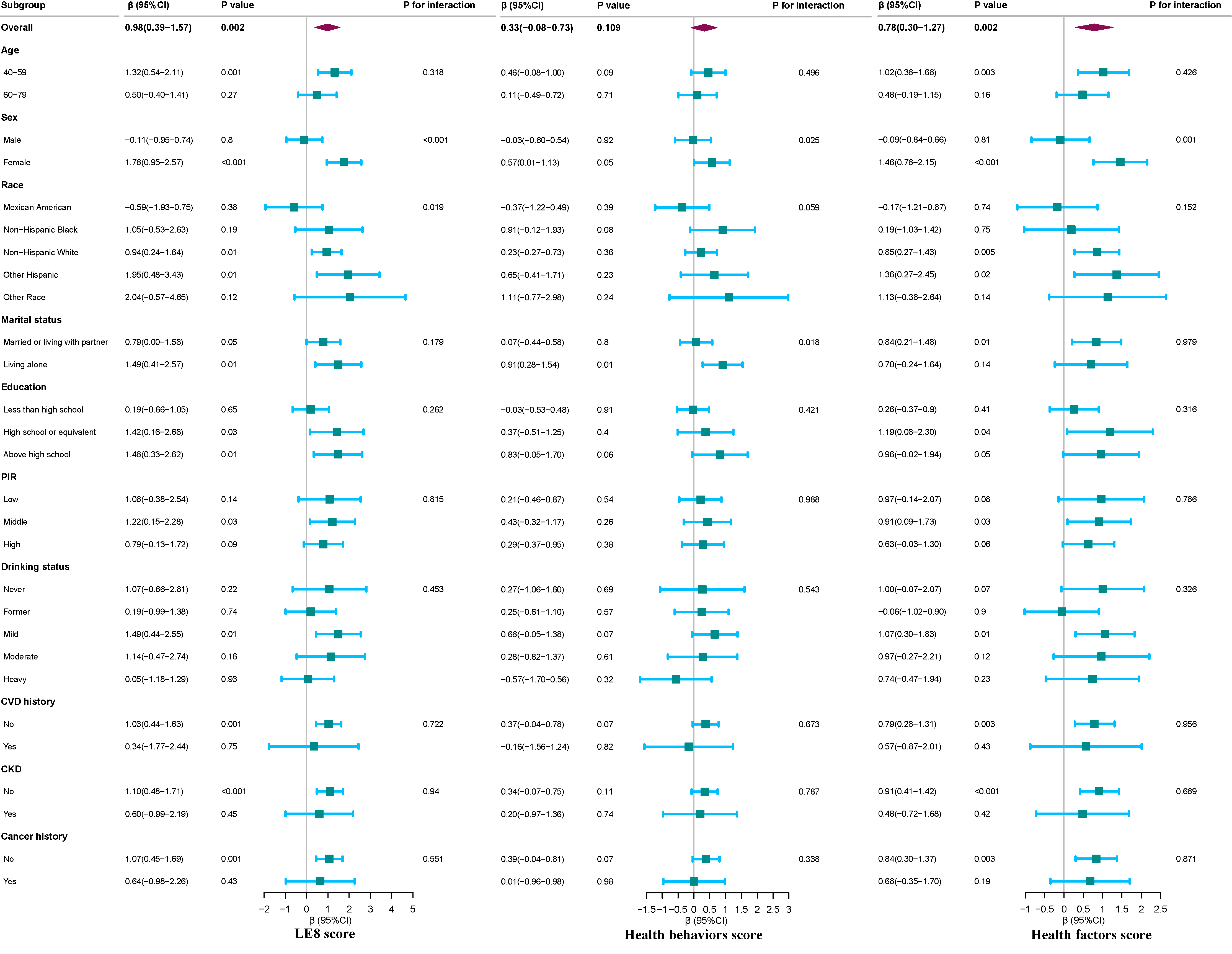

Supplement: Supplementary file 1 [file Image1.tif]
