# Supplementary material for: Sex differences in the association between Life’s Essential 8 and serum anti-aging Klotho protein levels: a cross-sectional analysis in middle-aged to older adults
Source: Front Aging. 2025 May 30;6:1458571. doi: 10.3389/fragi.2025.1458571 (PMC12162483; doi:10.3389/fragi.2025.1458571)
Supplement: Supplementary file 2 [file DataSheet1.docx]

Supplementary Material

Sex differences in the association between life's essential 8 and serum anti-aging Klotho protein levels: A cross-sectional analysis in middle-aged to older adults

Jing Zeng*, Tingting Zhang*, Yan Yang*, Jinjing Wang*, Dan Zheng, Yanwei Hou, Ye Tong, Xiaojing Fan, Qiaomin Wang

*** Correspondence:** Yi Fang: [fangyi5zhongxin@163.com](mailto:fangyi5zhongxin@163.com)

*** Correspondence:** Haoxian Tang: [19hxtang@stu.edu.cn](mailto:19hxtang@stu.edu.cn)

*** Correspondence:** Xuan Wang: [endocrine@163.com](mailto:endocrine@163.com)

**Supplementary Table 1** Baseline characteristics stratified by CVH score levels in weighted ^a^

| **Characteristic** | **Overall**  N = 9534 | **Low CVH**  N = 1592 | **Moderate CVH**  N = 6552 | **High CVH**  N = 1390 | ***p* value** |
| --- | --- | --- | --- | --- | --- |
| Age, Mean (SE) | 56.28 (0.18) | 57.83 (0.36) | 56.50 (0.19) | 54.46 (0.38) | <0.001 |
| Age, n (%) |  |  |  |  | <0.001 |
| 40–59 | 5,143 (62.45%) | 748 (55.57%) | 3,495 (61.81%) | 900 (69.33%) |  |
| 60–79 | 4,391 (37.55%) | 844 (44.43%) | 3,057 (38.19%) | 490 (30.67%) |  |
| Sex, n (%) |  |  |  |  | <0.001 |
| Male | 4,588 (47.22%) | 732 (44.01%) | 3,312 (50.04%) | 544 (39.45%) |  |
| Female | 4,946 (52.78%) | 860 (55.99%) | 3,240 (49.96%) | 846 (60.55%) |  |
| Race/ethnicity, n (%)^b^ |  |  |  |  | <0.001 |
| Mexican American | 1,356 (5.50%) | 222 (5.94%) | 1,005 (6.09%) | 129 (3.12%) |  |
| Non-Hispanic Black | 995 (3.93%) | 169 (4.90%) | 699 (4.01%) | 127 (2.99%) |  |
| Non-Hispanic White | 4,599 (77.14%) | 721 (71.03%) | 3,085 (76.25%) | 793 (84.36%) |  |
| Other Hispanic | 1,846 (8.34%) | 420 (13.82%) | 1,278 (8.63%) | 148 (3.62%) |  |
| Other Race ^c^ | 738 (5.10%) | 60 (4.30%) | 485 (5.02%) | 193 (5.92%) |  |
| Marital status, n (%) |  |  |  |  | <0.001 |
| Married or living with partner | 6,237 (71.41%) | 891 (61.68%) | 4,318 (70.80%) | 1,028 (80.14%) |  |
| Living alone | 3,297 (28.59%) | 701 (38.32%) | 2,234 (29.20%) | 362 (19.86%) |  |
| Education level, n (%) |  |  |  |  | <0.001 |
| Less than high school | 4,458 (36.49%) | 1,006 (57.17%) | 3,119 (38.47%) | 333 (15.58%) |  |
| High school or equivalent | 2,716 (30.75%) | 425 (30.35%) | 1,957 (32.73%) | 334 (24.04%) |  |
| Above high school | 2,360 (32.77%) | 161 (12.48%) | 1,476 (28.80%) | 723 (60.38%) |  |
| PIR, n (%) |  |  |  |  | <0.001 |
| Low | 2,668 (15.68%) | 670 (30.30%) | 1,807 (15.59%) | 191 (6.17%) |  |
| Middle | 3,461 (32.85%) | 628 (39.90%) | 2,446 (34.51%) | 387 (22.26%) |  |
| High | 3,405 (51.47%) | 294 (29.80%) | 2,299 (49.90%) | 812 (71.57%) |  |
| Drinking status, n (%) |  |  |  |  | <0.001 |
| Never | 1,249 (9.61%) | 178 (8.77%) | 880 (9.79%) | 191 (9.58%) |  |
| Former | 2,071 (17.88%) | 514 (30.92%) | 1,397 (17.75%) | 160 (9.58%) |  |
| Mild | 3,426 (40.70%) | 436 (31.05%) | 2,306 (39.06%) | 684 (52.95%) |  |
| Moderate | 1,370 (16.81%) | 183 (11.75%) | 951 (17.05%) | 236 (19.36%) |  |
| Heavy | 1,418 (15.00%) | 281 (17.52%) | 1,018 (16.36%) | 119 (8.53%) |  |
| CVD history, n (%)^d^ |  |  |  |  | <0.001 |
| No | 8,291 (89.59%) | 1,180 (76.87%) | 5,784 (90.19%) | 1,327 (96.06%) |  |
| Yes | 1,243 (10.41%) | 412 (23.13%) | 768 (9.81%) | 63 (3.94%) |  |
| CKD, n (%) |  |  |  |  | <0.001 |
| No | 8,030 (87.53%) | 1,132 (75.99%) | 5,589 (87.82%) | 1,309 (94.26%) |  |
| Yes | 1,504 (12.47%) | 460 (24.01%) | 963 (12.18%) | 81 (5.74%) |  |
| Cancer history, n (%) |  |  |  |  | 0.15 |
| No | 8,333 (85.79%) | 1,411 (86.79%) | 5,692 (85.15%) | 1,230 (87.38%) |  |
| Yes | 1,201 (14.21%) | 181 (13.21%) | 860 (14.85%) | 160 (12.62%) |  |
| LE8 score, Mean (SE) | 66.44 (0.34) | 41.89 (0.22) | 65.48 (0.16) | 86.37 (0.16) | <0.001 |
| Health behaviors score, Mean (SE) | 67.49 (0.45) | 39.65 (0.54) | 67.13 (0.31) | 87.50 (0.27) | <0.001 |
| Diet score, Mean (SE) | 42.75 (0.64) | 21.37 (0.71) | 39.49 (0.66) | 68.60 (0.79) | <0.001 |
| PA score, Mean (SE) | 70.85 (0.78) | 25.34 (1.38) | 72.51 (0.77) | 95.67 (0.49) | <0.001 |
| Nicotine exposure score, Mean (SE) | 72.24 (0.68) | 43.90 (1.50) | 72.21 (0.74) | 91.44 (0.65) | <0.001 |
| Sleep health score, Mean (SE) | 84.13 (0.37) | 68.00 (0.86) | 84.33 (0.31) | 94.29 (0.37) | <0.001 |
| Health factors score, Mean (SE) | 65.39 (0.34) | 44.12 (0.51) | 63.82 (0.28) | 85.24 (0.35) | <0.001 |
| BMI score, Mean (SE) | 57.92 (0.55) | 31.96 (1.03) | 55.69 (0.58) | 83.26 (0.74) | <0.001 |
| Blood lipids score, Mean (SE) | 58.39 (0.44) | 41.84 (1.02) | 56.60 (0.50) | 75.84 (0.94) | <0.001 |
| Blood glucose score, Mean (SE) | 81.51 (0.45) | 57.17 (0.94) | 81.95 (0.41) | 96.34 (0.40) | <0.001 |
| BP score, Mean (SE) | 63.74 (0.49) | 45.50 (1.04) | 61.03 (0.54) | 85.53 (0.68) | <0.001 |
| Klotho, Mean (SE), (pg/mL) | 842.39 (5.48) | 821.54 (9.35) | 833.84 (6.63) | 886.53 (9.91) | <0.001 |

^a^ All means and SEs for continuous variables and percentages for categorical variables were weighted. ^b^Race and ethnicity were self-reported. ^c^ Includes multiracial participants. CVH, cardiovascular health score; PIR, poverty income ratio; CVD, cardiovascular disease. ^d^ Includes congestive heart failure, coronary heart disease, angina, heart attack, and stroke.; CKD, chronic kidney disease; LE8, life’s essential 8; SE, standard error; PA, physical activity; BMI, body mass index; BP, blood pressure.

**Supplementary Table 2** Baseline characteristics stratified by sex in weighted, imputed data using multiple imputation ^a^

| **Characteristic** | **Overall**  **N =10781** | **Male**  **N = 5140** | **Female**  **N = 5641** | **p-value** |
| --- | --- | --- | --- | --- |
| Age, Mean (SE) | 56.33 (0.17) | 56.24 (0.20) | 56.42 (0.19) | 0.38 |
| Age, n (%) |  |  |  | 0.63 |
| 40–59 | 5,845 (62.20%) | 2,722 (62.46%) | 3,123 (61.97%) |  |
| 60–79 | 4,936 (37.80%) | 2,418 (37.54%) | 2,518 (38.03%) |  |
| Race/ethnicity, n (%)^b^ |  |  |  | 0.037 |
| Mexican American | 1,590 (5.86%) | 758 (6.07%) | 832 (5.68%) |  |
| Non-Hispanic Black | 1,213 (4.38%) | 532 (4.32%) | 681 (4.42%) |  |
| Non-Hispanic White | 5,008 (75.82%) | 2,421 (76.41%) | 2,587 (75.30%) |  |
| Other Hispanic | 2,104 (8.65%) | 1,001 (7.92%) | 1,103 (9.29%) |  |
| Other Race ^c^ | 866 (5.29%) | 428 (5.27%) | 438 (5.31%) |  |
| Marital status, n (%) |  |  |  | <0.001 |
| Married or living with partner | 7,046 (71.38%) | 3,774 (77.91%) | 3,272 (65.63%) |  |
| Living alone | 3,735 (28.62%) | 1,366 (22.09%) | 2,369 (34.37%) |  |
| Education level, n (%) |  |  |  | <0.001 |
| Less than high school | 5,173 (37.22%) | 2,518 (38.02%) | 2,655 (36.51%) |  |
| High school or equivalent | 3,008 (30.46%) | 1,313 (28.11%) | 1,695 (32.54%) |  |
| Above high school | 2,600 (32.32%) | 1,309 (33.87%) | 1,291 (30.95%) |  |
| PIR, n (%) |  |  |  | <0.001 |
| Low | 3,117 (16.71%) | 1,396 (15.48%) | 1,721 (17.80%) |  |
| Middle | 3,910 (32.98%) | 1,823 (31.19%) | 2,087 (34.55%) |  |
| High | 3,754 (50.31%) | 1,921 (53.33%) | 1,833 (47.65%) |  |
| Drinking status, n (%) |  |  |  | <0.001 |
| Never | 1,476 (10.17%) | 342 (5.70%) | 1,134 (14.10%) |  |
| Former | 2,343 (18.11%) | 1,196 (19.40%) | 1,147 (16.96%) |  |
| Mild | 3,809 (40.30%) | 2,077 (44.97%) | 1,732 (36.20%) |  |
| Moderate | 1,532 (16.44%) | 552 (11.55%) | 980 (20.74%) |  |
| Heavy | 1,621 (14.98%) | 973 (18.37%) | 648 (11.99%) |  |
| CVD history, n (%)^d^ |  |  |  | <0.001 |
| No | 9,367 (89.51%) | 4,317 (86.93%) | 5,050 (91.77%) |  |
| Yes | 1,414 (10.49%) | 823 (13.07%) | 591 (8.23%) |  |
| CKD, n (%) |  |  |  | 0.14 |
| No | 9,060 (87.32%) | 4,294 (88.00%) | 4,766 (86.72%) |  |
| Yes | 1,721 (12.68%) | 846 (12.00%) | 875 (13.28%) |  |
| Cancer history, n (%) |  |  |  | 0.31 |
| No | 9,457 (85.94%) | 4,509 (86.42%) | 4,948 (85.51%) |  |
| Yes | 1,324 (14.06%) | 631 (13.58%) | 693 (14.49%) |  |
| LE8, Mean (SE) | 66.44 (0.32) | 65.76 (0.32) | 67.03 (0.41) | <0.001 |
| CVH3, n (unweighted) (%) |  |  |  | <0.001 |
| Low | 1,791 (13.11%) | 815 (12.31%) | 976 (13.82%) |  |
| Moderate | 7,401 (67.52%) | 3,710 (71.54%) | 3,691 (63.98%) |  |
| High | 1,589 (19.37%) | 615 (16.15%) | 974 (22.21%) |  |
| Health behaviors score, Mean (SE) | 67.38 (0.43) | 67.26 (0.46) | 67.50 (0.54) | 0.46 |
| Diet score, Mean (SE) | 43.02 (0.63) | 40.79 (0.71) | 44.98 (0.76) | <0.001 |
| PA score, Mean (SE) | 70.38 (0.75) | 75.37 (0.86) | 66.00 (1.00) | <0.001 |
| Nicotine exposure score, Mean (SE) | 72.31 (0.63) | 69.37 (0.84) | 74.91 (0.73) | <0.001 |
| Sleep health score, Mean (SE) | 83.82 (0.37) | 83.51 (0.41) | 84.10 (0.49) | 0.065 |
| Health factors score, Mean (SE) | 65.49 (0.32) | 64.27 (0.34) | 66.56 (0.44) | <0.001 |
| BMI score, Mean (SE) | 58.10 (0.53) | 57.72 (0.60) | 58.44 (0.77) | 0.12 |
| Blood lipids score, Mean (SE) | 58.64 (0.42) | 57.38 (0.59) | 59.74 (0.53) | 0.002 |
| Blood glucose score, Mean (SE) | 81.42 (0.43) | 79.99 (0.52) | 82.67 (0.55) | <0.001 |
| BP score, Mean (SE) | 63.80 (0.47) | 61.98 (0.68) | 65.41 (0.52) | <0.001 |
| Klotho, Mean (SE), (pg/mL) | 844.41 (5.18) | 824.00 (5.95) | 862.35 (6.59) | <0.001 |

^a^ All means and SEs for continuous variables and percentages for categorical variables were weighted. ^b^Race and ethnicity were self-reported. ^c^ Includes multiracial participants. SE, standard error; PIR, poverty income ratio; CVD, cardiovascular disease. ^d^ Includes congestive heart failure, coronary heart disease, angina, heart attack, and stroke; CKD, chronic kidney disease; LE8, life’s essential 8; CVH, cardiovascular health score; PA, physical activity; BMI, body mass index; BP, blood pressure.

**Supplementary Table 3** Association of Health Behaviors Scores with serum Klotho levels in females

| Variables | Model 1^a^ | | Model 2^b^ | | Model 3^c^ | |
| --- | --- | --- | --- | --- | --- | --- |
|  | β (95% CI) | *p* Value | β (95% CI) | *p* Value | β (95% CI) | *p* Value |
| **Diet score** | | | | | | |
| Per 10 points increase | 3.23 (-0.40- 6.85) | 0.08 | 2.96 (-0.89- 6.81) | 0.13 | 2.58 (-1.23- 6.40) | 0.18 |
| Low (0–49) | 1(Ref) |  | 1(Ref) |  | 1(Ref) |  |
| Moderate (50–79) | 14.64 (-13.07-42.35) | 0.296 | 18.67 (-9.28-46.62) | 0.187 | 18.06 (-9.80-45.91) | 0.2 |
| High (80–100) | 29.1 (1.03- 57.16) | 0.042 | 26.58 (-2.97-56.13) | 0.077 | 23.96 (-5.39-53.31) | 0.108 |
| Trend.test |  | 0.042 |  | 0.071 |  | 0.099 |
| **PA score** | | | | | | |
| Per 10 points increase | 2.49 (-0.13- 5.11) | 0.062 | 1.88 (-0.80- 4.56) | 0.167 | 1.6 (-1.07- 4.26) | 0.235 |
| Low (0–49) | 1(Ref) |  | 1(Ref) |  | 1(Ref) |  |
| Moderate (50–79) | -41.82 (-93.92-10.27) | 0.114 | -49.37 (-100.99-2.24) | 0.06 | -48.52 (-99.57-2.54) | 0.062 |
| High (80–100) | 24.11 (-0.34- 48.56) | 0.053 | 17.79 (-7.33-42.91) | 0.162 | 15.28 (-9.64-40.20) | 0.225 |
| Trend.test |  | 0.034 |  | 0.111 |  | 0.16 |
| **Nicotine exposure score** | | | | | | |
| Per 10 points increase | 5.02 (1.75- 8.30) | 0.003 | 2.44 (-1.13- 6.02) | 0.177 | 2.2 (-1.45- 5.85) | 0.233 |
| Low (0–49) | 1(Ref) |  | 1(Ref) |  | 1(Ref) |  |
| Moderate (50–79) | 18.12 (-19.71-55.95) | 0.343 | 19.75 (-18.78-58.27) | 0.31 | 18.25 (-19.95-56.44) | 0.343 |
| High (80–100) | 46.32 (14.42- 78.22) | 0.005 | 20.18 (-14.20-54.56) | 0.245 | 17.69 (-17.38-52.77) | 0.317 |
| Trend.test |  | 0.002 |  | 0.312 |  | 0.392 |
| **Sleep health score** | | | | | | |
| Per 10 points increase | -1.33 (-5.19- 2.52) | 0.493 | -0.13 (-4.36- 4.10) | 0.952 | -0.53 (-4.80- 3.73) | 0.804 |
| Low (0–49) | 1(Ref) |  | 1(Ref) |  | 1(Ref) |  |
| Moderate (50–79) | 18.16 (-12.60-48.91) | 0.243 | 15.66 (-16.30-47.63) | 0.331 | 12.2 (-20.13-44.52) | 0.453 |
| High (80–100) | -5.07 (-32.34-22.19) | 0.712 | 2.22 (-27.56-32.00) | 0.882 | -1.1 (-31.14-28.94) | 0.942 |
| Trend.test |  | 0.339 |  | 0.813 |  | 0.678 |

a Crude model.

b Adjusted for age, sex, race/ethnicity, marital status, educational level, PIR and drinking status,

c Adjusted for age, sex, race/ethnicity, marital status, educational level, PIR, drinking status, CVD history，CKD and cancer history.

**Supplementary Table 4** Association of Health Factors Scores with serum Klotho levels in females

| Variables | Model 1^a^ | | Model 2^b^ | | Model 3^c^ | |
| --- | --- | --- | --- | --- | --- | --- |
|  | β (95% CI) | *p* Value | β (95% CI) | *p* Value | β (95% CI) | *p* Value |
| **BMI score** | | | | | | |
| Per 10 points increase | 5.11 (1.91- 8.31) | 0.002 | 6.23 (2.76- 9.69) | <0.001 | 5.79 (2.30- 9.28) | 0.002 |
| Low (0–49) | 1(Ref) |  | 1(Ref) |  | 1(Ref) |  |
| Moderate (50–79) | 17.03 (-7.15-41.21) | 0.165 | 25.4 (1.04- 49.76) | 0.041 | 21.96 (-2.77- 46.68) | 0.081 |
| High (80–100) | 47.25 (18.07-76.43) | 0.002 | 53.76 (22.23-85.29) | 0.001 | 51.33 (19.97- 82.68) | 0.002 |
| Trend.test |  | 0.002 |  | <0.001 |  | 0.002 |
| **Blood lipids score** | | | | | | |
| Per 10 points increase | 9.45 (5.63- 13.26) | <0.001 | 7.32 (3.48- 11.15) | <0.001 | 7.32 (3.50- 11.14) | <0.001 |
| Low (0–49) | 1(Ref) |  | 1(Ref) |  | 1(Ref) |  |
| Moderate (50–79) | 62.6 (31.27- 93.93) | <0.001 | 52.78 (22.05-83.52) | 0.001 | 50.36 (19.26- 81.47) | 0.002 |
| High (80–100) | 53.79 (26.26-81.32) | <0.001 | 42.78 (15.25-70.31) | 0.003 | 43.74 (16.31- 71.16) | 0.002 |
| Trend.test |  | <0.001 |  | 0.002 |  | 0.002 |
| **Blood glucose score** | | | | | | |
| Per 10 points increase | 2.04 (-1.85- 5.93) | 0.3 | 1.45 (-2.66- 5.55) | 0.484 | 0.21 (-3.90- 4.31) | 0.92 |
| Low (0–49) | 1(Ref) |  | 1(Ref) |  | 1(Ref) |  |
| Moderate (50–79) | 16.43 (-19.45-52.32) | 0.365 | 24.96 (-12.11-62.02) | 0.183 | 16.08 (-22.29-54.44) | 0.405 |
| High (80–100) | 28.57 (-2.53-59.67) | 0.071 | 29.29 (-3.60- 62.18) | 0.08 | 19.3 (-14.69- 53.30) | 0.261 |
| Trend.test |  | 0.046 |  | 0.093 |  | 0.27 |
| **BP score** | | | | | | |
| Per 10 points increase | 8.09 (4.29- 11.90) | <0.001 | 5.98 (1.94- 10.02) | 0.004 | 5.32 (1.20- 9.44) | 0.012 |
| Low (0–49) | 1(Ref) |  | 1(Ref) |  | 1(Ref) |  |
| Moderate (50–79) | 16.22 (-10.96-43.41) | 0.238 | 8.34 (-19.99- 36.67) | 0.558 | 2.73 (-26.53- 31.99) | 0.852 |
| High (80–100) | 58.79 (27.39-90.19) | <0.001 | 43.06 (10.82-75.29) | 0.01 | 38.33 (5.82- 70.83) | 0.022 |
| Trend.test |  | <0.001 |  | 0.005 |  | 0.009 |

a Crude model.

b Adjusted for age, sex, race/ethnicity, marital status, educational level, PIR and drinking status,

c Adjusted for age, sex, race/ethnicity, marital status, educational level, PIR, drinking status, CVD history，CKD and cancer history.

**Supplementary Table 5** Association of LE8, Health Behaviors, and Health Factors Score with serum Klotho levels in females: analysis using multiple imputation

| Variables | | Model 1^a^ | | | | Model 2^b^ | | | Model 3^c^ | |
| --- | --- | --- | --- | --- | --- | --- | --- | --- | --- | --- |
|  |  | β (95% CI) | | *p* Value | | β (95% CI) | *p* Value | | β (95% CI) | *p* Value |
| **LE8 score** | | | | | | | | | | |
| Per 10 points increase | | 21.46 (14.73-28.20) | | <0.001 | | 20.31 (13.04-27.59) | <0.001 | | 18.94 (11.42- 26.47) | <0.001 |
| Low CVH | | 1(Ref) | |  | | 1(Ref) |  | | 1(Ref) |  |
| Moderate CVH | | 29.18 (0.42-57.94) | | 0.047 | | 29.34 (1.27-57.41) | 0.041 | | 24.45 (-3.98- 52.88) | 0.091 |
| High CVH | | 97.19 (63.03-131.35) | | <0.001 | | 88.08 (53.21-122.95) | <0.001 | | 81.08 (45.15- 117.01) | <0.001 |
| Trend.test | |  | | <0.001 | |  | <0.001 | |  | <0.001 |
| **Health behaviors score** | | | | | | | | | | |
| Per 10 points increase | 8.28 (3.63- 12.93) | | <0.001 | | 6.62 (1.50- 11.74) | | 0.012 | 5.69 (0.53- 10.85) | | 0.031 |
| Low (0–49) | 1(Ref) | |  | | 1(Ref) | |  | 1(Ref) | |  |
| Moderate (50–79) | 13.46 (-9.19- 36.11) | | 0.24 | | 7.59 (-16.45- 31.63) | | 0.53 | 4.16 (-19.94- 28.27) | | 0.731 |
| High (80–100) | 52.32 (24.79- 79.85) | | <0.001 | | 44.43 (13.86-75.00) | | 0.005 | 39.01 (8.32- 69.71) | | 0.014 |
| Trend.test |  | | <0.001 | |  | | 0.003 |  | | 0.007 |
| **Health factors score** | |  | |  | |  |  | |  |  |
| Per 10 points increase | | 18.16 (12.06-24.26) | | <0.001 | | 17.13 (10.80-23.45) | <0.001 | | 16.25 (9.75- 22.75) | <0.001 |
| Low (0–49) | | 1(Ref) | |  | | 1(Ref) |  | | 1(Ref) |  |
| Moderate (50–79) | | 6.51 (-12.04-25.06) | | 0.487 | | 8.87 (-9.44- 27.18) | 0.337 | | 3.96 (-15.26- 23.18) | 0.682 |
| High (80–100) | | 86.18 (55.82-116.55) | | <0.001 | | 79.45 (48.18-110.73) | <0.001 | | 74.67 (42.88- 106.47) | <0.001 |
| Trend.test | |  | | <0.001 | |  | <0.001 | |  | <0.001 |

^a^ Crude model.

^b^ Adjusted for age, race/ethnicity, marital status, educational level, PIR and drinking status,

^c^ Adjusted for age, race/ethnicity, marital status, educational level, PIR, drinking status, CVD history, CKD and cancer history.

**Supplementary Table 6** Association of LE8, Health Behaviors, and Health Factors Score with serum Klotho levels in males: analysis using multiple imputation

| Variables | Model 1^a^ | | Model 2^b^ | | | Model 3^c^ | |
| --- | --- | --- | --- | --- | --- | --- | --- |
|  | β (95% CI) | *p* Value | β (95% CI) | *p* Value | | β (95% CI) | *p* Value |
| **LE8 score** | | | | | | | |
| Per 10 points increase | 3.71 (-3.30- 10.72) | 0.296 | 0.3 (-7.53- 8.12) | | 0.94 | -0.29 (-8.23- 7.64) | 0.941 |
| Low CVH | 1(Ref) |  | 1(Ref) | |  | 1(Ref) |  |
| Moderate CVH | 4.86 (-26.44-36.16) | 0.758 | 2.64 (-28.93-34.22) | | 0.868 | 1.51 (-29.37- 32.38) | 0.923 |
| High CVH | 23.71 (-9.11-56.52) | 0.154 | 10.8 (-24.37-45.96) | | 0.542 | 8.75 (-26.42- 43.91) | 0.621 |
| Trend.test |  | 0.141 |  | | 0.531 |  | 0.608 |
| **Health behaviors score** | | | | | | | |
| Per 10 points increase | 1.55 (-3.61- 6.72) | 0.551 | 0.04 (-5.51- 5.60) | 0.987 | | -0.07 (-5.67- 5.53) | 0.981 |
| Low (0–49) | 1(Ref) |  | 1(Ref) |  | | 1(Ref) |  |
| Moderate (50–79) | -5.77 (-35.36-23.82) | 0.699 | -9.88 (-39.72-19.95) | 0.51 | | -9.95 (-39.63-19.73) | 0.505 |
| High (80–100) | 8.18 (-19.94- 36.29) | 0.564 | 0.25 (-29.67-30.17) | 0.987 | | -0.46 (-30.57- 29.65) | 0.976 |
| Trend.test |  | 0.426 |  | 0.835 | |  | 0.877 |
| **Health factors score** |  |  |  |  | |  |  |
| Per 10 points increase | 2.82 (-4.19- 9.83) | 0.425 | 0.3 (-6.88- 7.47) | 0.934 | | -0.27 (-7.59- 7.05) | 0.942 |
| Low (0–49) | 1(Ref) |  | 1(Ref) |  | | 1(Ref) |  |
| Moderate (50–79) | 0.04 (-29.15-29.23) | 0.998 | -4.26 (-33.55-25.04) | 0.773 | | -6.45 (-36.34-23.43) | 0.667 |
| High (80–100) | 7.42 (-31.98-46.82) | 0.709 | -5.56 (-44.93-33.80) | 0.779 | | -8.54 (-48.67- 31.59) | 0.672 |
| Trend.test |  | 0.706 |  | 0.779 | |  | 0.673 |

^a^ Crude model.

^b^ Adjusted for age, race/ethnicity, marital status, educational level, PIR and drinking status,

^c^ Adjusted for age, race/ethnicity, marital status, educational level, PIR, drinking status, CVD history, CKD and cancer history.

**Supplementary Table 7** Association of LE8, Health Behaviors, and Health Factors Score with serum Klotho levels in all participants included

| Variables | | Model 1^a^ | | | | Model 2^b^ | | | | Model 3^c^ | |
| --- | --- | --- | --- | --- | --- | --- | --- | --- | --- | --- | --- |
|  |  | β (95% CI) | | *p* Value | | β (95% CI) | | *p* Value | | β (95% CI) | *p* Value |
| **LE8 score** | | | | | | | | | | | |
| Per 10 points increase | | 14.13 (9.08–19.18) | | <0.001 | | 11.23 (5.35–17.12) | | <0.001 | | 9.81 (3.88–15.75) | 0.002 |
| Low CVH | | 1(Ref) | |  | | 1(Ref) | |  | | 1(Ref) |  |
| Moderate CVH | | 12.3 (-8.11–32.71) | | 0.234 | | 13.74 (-7.97–35.45) | | 0.211 | | 9.51 (-11.83–30.86) | 0.376 |
| High CVH | | 64.99 (40.19–89.80) | | <0.001 | | 52.57 (24.21–80.93) | | <0.001 | | 46.33 (17.91–74.74) | 0.002 |
| Trend.test | |  | | <0.001 | |  | | <0.001 | |  | 0.001 |
| **Health behaviors score** | | | | | | | | | | | |
| Per 10 points increase | 5.57 (1.98– 9.17) | | 0.003 | | 3.8 (-0.27– 7.87) | | 0.067 | | 3.26 (-0.75– 7.27) | | 0.109 |
| Low (0–49) | 1(Ref) | |  | | 1(Ref) | |  | | 1(Ref) | |  |
| Moderate (50–79) | 3.04 (-15.94–22.02) | | 0.751 | | 0.22 (-20.10–20.54) | | 0.983 | | -1.28 (-21.33–18.78) | | 0.899 |
| High (80–100) | 34.58 (14.59–54.57) | | <0.001 | | 26.09 (3.82–48.36) | | 0.022 | | 22.9 (0.79– 45.00) | | 0.043 |
| Trend.test |  | | <0.001 | |  | | 0.009 | |  | | 0.018 |
| **Health factors score** | |  | |  | |  | |  | |  |  |
| Per 10 points increase | | 11.56 (6.89– 16.23) | | <0.001 | | 9.02 (4.22– 13.83) | | <0.001 | | 7.84 (3.00– 12.69) | 0.002 |
| Low (0–49) | | 1(Ref) | |  | | 1(Ref) | |  | | 1(Ref) |  |
| Moderate (50–79) | | 0.02 (-16.70– 16.74) | | 0.998 | | -0.82 (-17.32– 15.68) | | 0.921 | | -6.65 (-23.82– 10.51) | 0.441 |
| High (80–100) | | 53.23 (29.26– 77.20) | | <0.001 | | 39.66 (15.78– 63.54) | | 0.002 | | 33.2 (9.02– 57.38) | 0.008 |
| Trend.test | |  | | <0.001 | |  | | 0.001 | |  | 0.005 |

^a^ Crude model.

^b^ Adjusted for age, sex, race/ethnicity, marital status, educational level, PIR and drinking status,

^c^ Adjusted for age, sex, race/ethnicity, marital status, educational level, PIR, drinking status, CVD history, CKD and cancer history.

**Supplementary Figure 1** Subgroup analyses of the association between LE8, Health Behaviors, and Health Factors Score and serum Klotho levels in all study participants Except for the stratification component itself, each stratification factor was adjusted for all other variables (sex, age, race, education level, family income, smoking status, drinking status, physical activity, UA, TC, LDL-C, diabetes and CVD).
